# Impact of RNA degradation on gene expression profiling

**DOI:** 10.1186/1755-8794-3-36

**Published:** 2010-08-09

**Authors:** Lennart Opitz, Gabriela Salinas-Riester, Marian Grade, Klaus Jung, Peter Jo, Georg Emons, B Michael Ghadimi, Tim Beißbarth, Jochen Gaedcke

**Affiliations:** 1 DNA Microarray Facility, University Medicine Göttingen, Humboldtallee 23, 37073 Göttingen, Germany; 2 Department Surgery, University Medicine Göttingen, Robert-Koch-Str. 40, 37073 Göttingen, Germany; 3 Department Medical Statistics, University Medicine Göttingen, Humboldtallee 32, 37073 Göttingen, Germany

## Abstract

**Background:**

Gene expression profiling is a highly sensitive technique which is used for profiling tumor samples for medical prognosis. RNA quality and degradation influence the analysis results of gene expression profiles. The impact of this influence on the profiles and its medical impact is not fully understood. As patient samples are very valuable for clinical studies, it is necessary to establish criteria for the RNA quality to be able to use these samples in later analysis.

**Methods:**

To investigate the effects of RNA integrity on gene expression profiling, whole genome expression arrays were used. We used tumor biopsies from patients diagnosed with locally advanced rectal cancer. To simulate degradation, the isolated total RNA of all patients was subjected to heat-induced degradation in a time-dependent manner. Expression profiling was then performed and data were analyzed bioinformatically to assess the differences.

**Results:**

The differences introduced by RNA degradation were largely outweighed by the biological differences between the patients. Only a relatively small number of probes (275 out of 41,000) show a significant effect due to degradation. The genes that show the strongest effect due to RNA degradation were, especially, those with short mRNAs and probe positions near the 5' end.

**Conclusions:**

Degraded RNA from tumor samples (RIN > 5) can still be used to perform gene expression analysis. A much higher biological variance between patients is observed compared to the effect that is imposed by degradation of RNA. Nevertheless there are genes, very short ones and those with the probe binding side close to the 5' end that should be excluded from gene expression analysis when working with degraded RNA. These results are limited to the Agilent 44 k microarray platform and should be carefully interpreted when transferring to other settings.

## Background

High-throughput microarray technology is ideally suited for analyzing thousands of genes in a single experiment and allows a better understanding of the molecular mechanisms of cancer development and progression. Gene expression arrays have a profound influence on the development of new diagnostic and therapeutic strategies, such as the prediction of prognosis in breast cancer [1] or the response to a preoperative radio-chemotherapy (RT/CT) in rectal cancer [2]. High-quality RNA is considered a prerequisite for high-throughput analysis. Nevertheless, RNA degradation is a critical problem in all experimental settings and for clinical samples in particular. Additionally, the investigation of clinical samples poses a second problem. Due to the heterogeneity of tumor samples, a high number of patients is needed for statistical analysis. There is thus an ongoing debate as to how far gene expression results are affected by various degrees of degradation [3-6] and to what extent of degradation the tissue samples with poor RNA quality can be included in an analysis.

Degradation of RNA itself is a physiological process during the cell cycle to regulate RNA-dependent mechanisms. Many intracellular enzymes, such as ribonucleases, (endo- and exonucleases), as well as other cofactors, are involved and exhibit prevalent activity in all organisms of life [7]. The multiplicity of functions that characterize ribonucleases in eukaryotes underlines the key importance of mechanisms that specifically not only target and degrade RNAs but also function in RNA-processing reactions and presumably enhance the overall efficiency of degradation pathways [8,9]. Although these processes were well investigated in the past and the level of knowledge is increasing steadily, little is known as to how far the mechanisms can be translated into cells that have been taken out of the organism as is done when biopsies are taken.

Moreover, beside RNA-degrading enzymes and cofactors, tissue-specific factors such as the extent of necrosis and storage conditions have to be considered to avoid degradation of RNA. In the past, liquid nitrogen was considered to be the standard method for archiving tissue samples. This, however, poses numerous logistical problems surrounding the provision of nitrogen and the transportation of frozen tissue samples. This is essentially a problem for clinical trials, in which patients are frequently recruited in non-university hospitals where access to liquid nitrogen cannot be guaranteed. Therefore, preserving liquids have been developed and studied with respect to RNA stability [10-12].

To assess RNA quality, different methods have been applied [5,13]. The electrophoretic-based generation of a RNA integrity number (RIN) [14] using Agilent's Bioanalyzer provides a user-independent method of reproducibly assessing degradation of RNA. In the recently published literature comparing different levels of RNA integrity, the RIN has frequently been used, thus allowing easy comparison of the postulated values that were indicated as thresholds for considering good and poor RNA quality [4,5]. Within these publications, tissue was treated with different temperature levels to achieve degradation. Interestingly, the influence of similar temperature levels resulted in very different degradation results. Furthermore this pre-isolation degradation more or less mimics a prolonged time to storage and initiates the complex process of cell hypoxia and consecutive cell death.

Here in this study, we aim to assess the influence of RNA degradation on gene expression and to analyze the systematic effect that is observable when using RNA with different RINs. Therefore, we performed gene expression analysis of tissues from tumor biopsies of several patients using gene expression microarrays. We performed degradation by heat-treatment of isolated RNA to avoid transcriptional processes in the context of cell death.

## Methods

### Samples and sample preparation

Tumor biopsies from three patients with locally advanced rectal cancer were taken prior to the preoperative radio-chemotherapy. These patients were treated within the CAO/ARO/AIO-04 trial (EudraCT-Number: 2006-002385-20), biopsies were taken according to the guidelines set by the Local Ethical Review Board (application number: 9/8/08) and patients gave written consent. Due to the rigid rectoscopy, biopsies were stored in RNAlater (Qiagen, Hilden, Germany) within 10 to 20 seconds after removal and subsequently kept at 5°C for 24 hours followed by storage at -20°C. Extraction of nucleic acids was carried out four months prior to the following experiments and was performed using TRIZOL (Invitrogen, Carlsbad, CA) following standard procedures as previously described [15] (details can be found at http://www.riedlab.nci.nih.gov/protocols.asp) and stored at -80°C.

### RNA Degradation and Quality Control

Heat-induced degradation was carried out at 60°C on a thermal block (Eppendorf Thermomixer). For each of the three patients, four different time points were defined for the analysis of degradation state, namely 0:00 h; 1:45 h; 2:30 h; and 3:15 h respectively. Time points and degradation temperature were chosen based on preliminary test results (data not shown) revealing that no and very low degradation occurred at room temperature and 45°C, respectively.

After thermal degradation, total RNA was checked for quantity, purity and integrity of the 18 S and 28 S ribosomal bands by capillary electrophoresis. RNA degradation was assessed using the Agilent 2100 Bioanalyzer following the manufacturer's standard protocol. In brief, after loading RNA Nano LabChip with the gel-dye-mix, each of the 12 measuring chambers was filled with RNA (concentration between 200-300 ng/*μ*l) and the provided marker. The measurement was then carried out by the Bioanalyzer and a separate RNA integrity number as well as the correlating electropherogram and a gel-like image was provided by the software for each of the samples. Each RNA sample was split between four different tubes. RNA quality was rated according to the RNA integrity number (RIN). Measurement of RIN was carried out prior to the array hybridization without freezing and thawing again.

### Microarrays

Microarrays were done using the "Low RNA Input linear Amplification Kit Plus, One Color" protocol (Agilent Technologies, Inc. 2007; Cat. N°: 5188-5339) and the Agilent RNA Spike-In Kit for One color (Agilent Technologies, Inc. 2007; Cat. N°: 5188-5282) following the manufacturer's standard protocol. Global gene expression analysis was applied using the Human 4 × 44 K design array from Agilent Technologies (G4112F). 600 ng of total RNA were used as a starting material to prepare cDNA. cDNA synthesis and in vitro transcription (IVT) were performed according to the manufacturer's recommendation. Quantity and efficiency of the labeled amplified cRNA were determined using the NanoDrop ND-1000 UV-VIS Spectrophotometer version 3.2.1. The hybridizations were performed for 17 hours at 10 rpm and 65°C in the Hybridization Oven (Agilent). Washing and staining of the arrays were done according to the manufacturer's recommendation. Cy3 intensities were detected by one-color scanning using an Agilent DNA microarray scanner (G2505B) at 5 micron resolution. Scanned image files were visually inspected for artifacts and then analyzed.

### Semi-quantitative Real-time PCR

One *μ*g of total RNA was reverse-transcribed in a 25 *μ*l reaction volume into first-strand cDNA using Superscript III and random hexamers (Invitrogen). Three *μ*l of cDNA mix was added to 12.5 *μ*l of i*Q*™*SY BR^® ^*Green Supermix (Bio-Rad Laboratories, Hercules, CA), and 375 ng primer solution to a total of 25 *μ*l per reaction. Amplification efficiency was assessed using LinRegPCR [16]. The corresponding primer sequences can be found in Table 1. Real-time PCR analysis was performed in a Bio-Rad iCycler iQ5 (Bio-Rad, Munich, Germany) using the following cycling parameters: 10 min at 95°C, 40 cycles of 15 sec at 95°C, one min at 60°C. The resulting Ct values were normalized based on the mean of three housekeeping genes, GAPDH, HPRT1 and YWHAZ.

**Table 1:** PCR Primers used for semi-quantitative real time PCR validation.

Systematic Name	Gene Name	Primer Sequence (5'→3')
NM_005185.2	CALML3	Fwd: AGGCCTTCTCCCTGTTTGAC
		Rev: CCGGTCGATCTCACTCATC
NM_018403.4	DCP1A	Fwd: AGCATCACCAGCAGATCCTT
		Rev: TATTCCACAGCCTTGCTCCT
NM_145301.2	FAM18B2	Fwd: AAGAGCCATTGGGTGTTTGA
		Rev: AGGCAATAAGTCCCAACCAA
NM_005336.3	HDLBP	Fwd: CAGGACCTGCTCCACTGTTT
		Rev: AGGCCAGAGTGCTGACTGAC
XR_038543.2	LOC644604	Fwd: TGCCTGGGTCTTGGATAAAC
		Rev: GGGCATCAACGATAGTCACA
NM_018222.3	PARVA	Fwd: TTTGAGCTCATGCAAGATGG
		Rev: CGGTACTTGGTGAAGAGGTTG
NM_001135771.1	RPN2	Fwd: GCTTCTGCTCTTCGCTCTGT
		Rev: CCAGCATAGCAGCATGTCC
NM_003133.5	SRP9	Fwd: CAGACCTGGGAGGAGTTCAG
		Rev: CACAAGTTCCCATCAGAATGC
NM_006082.2	TUBA1B	Fwd: ACGTGGTTCCCAAAGATGTC
		Rev: CACAGTGGGAGGCTGGTAGT

NM_002046.3	GAPDH	Fwd: CCACATCGCTCAGACACCAT
		Rev: CCAGGCGCCCAATACG
NM_000194.1	HPRT1	Fwd: TGACACTGGCAAAACAATGCA
		Rev: GGTCCTTTTCACCAGCAAGCT
NM_003406.2	YWHAZ	Fwd: ACTTTTGGTACATTGTGGCTTCAA
		Rev: CCGCCAGGACAAACCAGTAT

### Data Analysis

Intensity data were extracted using Agilent's Feature Extraction (FE) software (version 9.5.3.1) including a quality control based on internal controls using Agilent's protocol GE1_107_Sep09. All chips passed the quality control and were analyzed using the Limma package [17] of Bioconductor [18].

The data discussed in this paper are generated in compliance with the MIAME guidelines and have been deposited in NCBI's Gene Expression Omnibus and are accessible through GEO Series accession number GSE17753 http://www.ncbi.nlm.nih.gov/geo/query/acc.cgi?acc=GSE17753.

The microarray data analysis consists of the following steps:

1. between-array normalization, 2. global clustering and PCA-analysis, 3. fitting the data to a linear model, 4. detection of differential gene expression and 5. over-representation analysis of differentially expressed genes. Quantile-normalization was applied to the log2-transformed intensity values as a method for between-array normalization, to ensure that the intensities had similar distributions across arrays [19].

For cluster analysis, we used a hierarchical approach with the average linkage-method. Distances were measured as 1 - Pearson's Correlation Coefficient. The PCA was performed using the princomp-function in the R software. To estimate the average group values for each gene and assess differential gene expression, a simple linear model was fitted to the data, and group-value averages and standard deviations for each gene were obtained. To find genes with significant expression changes between groups, empirical Bayes statistics were applied to the data by moderating the standard errors of the estimated values [17].

P-values were obtained from the moderated t-statistic and corrected for multiple testing with the Benjamini-Hochberg method [20]. The p-value adjustment guarantees a smaller number of false positive findings by controlling the false discovery rate (fdr). For each gene, the null hypothesis, that there is no differential expression between degradation levels, was rejected when its fdr was lower than 0.05. To find over-represented functions (as represented by Gene Ontology terms [21]), we used the additional R package topGO [22].

The statistical significance of comparisons between different gene lists was examined with the nonparametric Wilcoxon rank sum test, using R. Bonferroni-corrected [23] p-values < 0.05 were considered significantly different.

To correlate RNA degradation time with RIN values from patient samples, we used Kendall's rank correlation coefficient *τ*.

## Results

### Effect of RNA degradation on comparability between patients

In order to analyze the effects of RNA degradation on gene expression profiles, we selected biopsies of real patient tissue. Specifically, pre-therapeutic biopsy tumor samples from three individual patients diagnosed with rectal cancer were used. The total RNA of each patient was degraded thermically in a time-dependent manner (see Methods). Four different time-points of degradation (Control: 0:00 h, TP1: 1:45 h, TP2: 2:30 h and TP3: 3:15 h) were selected.

**Figure 1:**
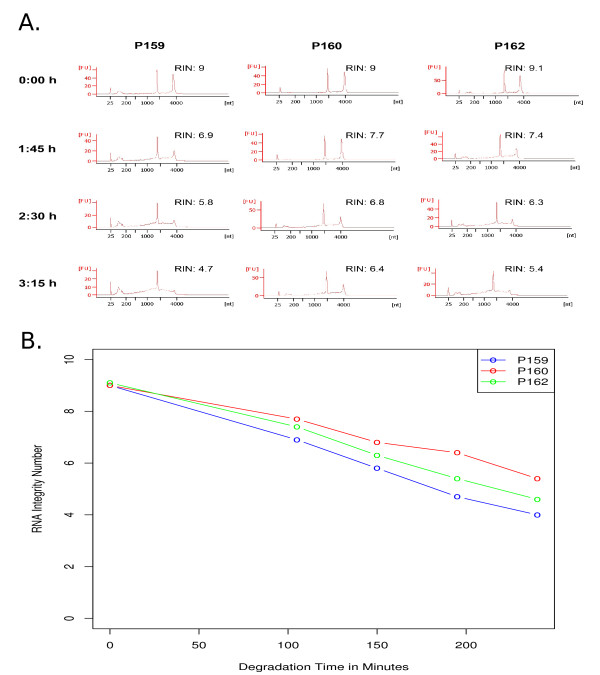
**Degradation of RNA**. The effect of degradation time on calculated RNA Integrity Numbers (RINs) for the given samples P159, P160 and P162 is shown. (**A**) Bioanalyzer profiles of total RNA for degradation levels used in the microarray study (control, 1:45 h, 2:30 h, 3:15 h). RINs are shown next to each total RNA profile. (**B**) RIN-dependence of degradation time separately for all patients. An additional time point (240 Min/4:00 h) without gene expression profile is also present.

To determine the integrity of the RNA starting materials for the microarray analysis at the different time-points, we evaluated the quality of each sample using the RNA integrity number (RIN). Using this tool, sample integrity is determined by the entire electrophoretic trace (Bioanalyzer profiles) of the RNA sample, including the presence or absence of degradation products. The Bioanalyzer profiles, as well as the calculated RINs of the samples, are shown in Figure 1A. The calculated RNA integrity numbers for all samples depending on degradation time are displayed in Figure 1B. A strong negative correlation between RIN-values and degradation time in all three patients is evident. The Null-Hypothesis that there is no negative correlation can be rejected using a statistical correlation test on Kendall's rank correlation (*τ *= -0:862, p-value = 2.530e-05).

**Figure 2:**
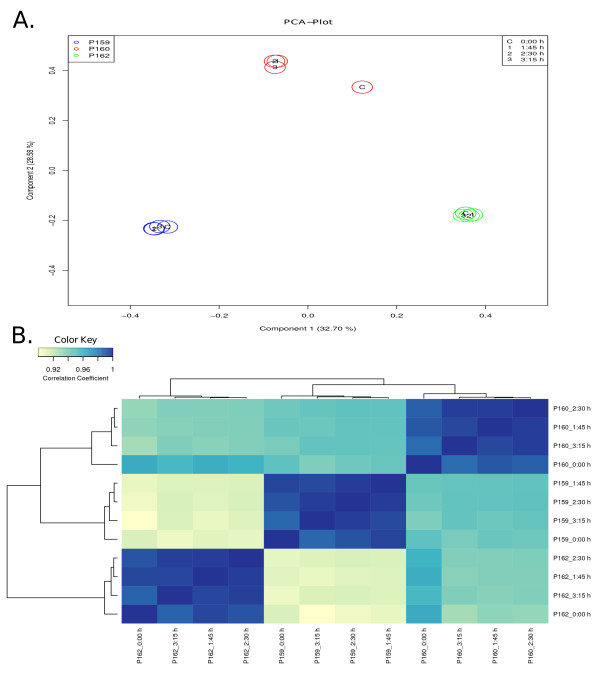
**Effect of RNA degradation on comparability between patients**. (**A**) Two-dimensional PCA plot of genome-wide expression profiles showing principal components 1 and 2. The first axis (PC1) accounts for 33% of the overall variance of the data, the second axis accounts for 29% (PC2). The colors blue (P159), red (P160) and green (P162) refer to the different patients. The degradation levels are represented by the following symbols: C (0:00 h), 1 (1:45 h), 2 (2:30 h) and 3 (3:15 h). (**B**) shows pairwise correlations between all samples of patients. The cells in the visualization are colored by Pearson's correlation coefficient values with deeper colors indicating higher positive (blue) correlations. The heatmap is flanked by clustering dendrograms showing the similarity between samples in a hierarchical approach.

Next, we analyzed the samples of the different patients at different RNA degradation levels using gene expression profiling. With this we address the question of how significant the influence of RNA degradation is on the gene expression profiles in comparison to the overall influence of different patients. To gain insights into the data obtained by the microarrays and, especially, to analyze how patient samples correlate with degradation stages, different bioinformatic methods were applied. The multivariate data analysis method PCA (principal components analysis) was used to visualize similarities or dissimilarities between genome-wide expression profiles of patient samples and degradation stages in a two-dimensional plot. PCA is a linear projection method that allows visualization of high-dimensional data in a lower dimensional space. The results of the PCA analysis for the normalized microarray dataset are shown in Figure 2A. The first axis of the plot (first principal component, PC1) accounts for 33% of the overall variance of the data, while the second axis accounts for 29% (PC2). It can clearly be seen that samples from the same patient are very similar to each other regardless of the degradation level. The differences between the different patients, which are to be analyzed, contribute the major part of the overall differences.

Furthermore, we applied a clustering approach using an agglomerative hierarchical clustering algorithm. Based on the hierarchical clustering, we observe that the samples which are derived from the same patient cluster together regardless of the degradation level. To demonstrate this, the pairwise similarity metrics between samples was calculated based on normalized expression measurements of all genes using Pearson's correlation coefficient. The hierarchical clustering was applied to the resulting matrix of pairwise correlation coefficients. The resulting correlation matrix is shown as a heatmap in Figure 2B. Similarly to the PCA-results, the correlations between samples of the same patient are much higher than the correlations between samples of different biological backgrounds.

In conclusion, both analysis methods, i.e. cluster analysis and PCA, provide strong evidence that differences between patients are larger and more significant than those observed by the thermal degradation of RNA and therefore comparison of reliable clinically relevant data of patient samples will not be hindered by the effects of RNA degradation for a realistic range of RIN values.

### Genes effected by RNA degradation

While the differences we observe between patients outweigh the differences we observe due to RNA degradation, we still wanted to analyze the genes which are affected by RNA degradation more closely. These are genes that will have to be carefully considered in studies where different patient groups are compared, because their expression might be influenced by the RNA quality rather than reflecting biological differences between the patient groups which are to be analyzed.

Analysis of differential gene expression was performed in order to identify those genes which are significantly affected by RNA degradation. The quantitative changes in gene expression with increasing RNA degradation levels were investigated and the change of expression level was further considered as level of representation, i.e. either over- or under-represented RNA species.

For the statistical analysis of differential expression, we defined four different groups of degradation (Control, TP1, TP2 and TP3) including three biological replicates (P159, P160 and P162) per group. Three comparisons were performed, namely TP1 against the control group (TP1 vs Control); TP2 against the control group (TP2 vs Control) and TP3 against the control group (TP3 vs Control). We considered all differentially expressed genes (DEGs) with a fdr of 5% and a fold change greater than 2-fold as significantly altered. The number of significant DEGs increases from 15 (TP1 vs Control), 73 (TP2 vs Control) to 275(TP3 vs Control) and is negatively correlated to the RIN values of the corresponding samples. Furthermore, we analyzed three groups of RNAs separately in each of the three comparisons: 1. under-represented as compared to the average RNA population; 2. over-represented or more-stable to the rest of the RNA and 3. normally-represented are the seemingly not-differential genes that are degraded at a similar level as the average RNA population. We also noted that the absolute signal intensity of the majority of genes before normalization decreases with increased time. Therefore these genes have to be considered as affected by degradation as well, however, this difference does not affect analysis due to normalization. The majority of DEGs is under-represented with the increasing RNA degradation of the samples as can be seen in Table 2.

**Table 2:** Summary of Gene Regulation

Comparison	#Over-Represented	#Under-Represented	#Normally-Represented
TP1 vs Control	11	4	40985
TP2 vs Control	22	51	40937
TP3 vs Control	65	210	40725

All genes are grouped by the type of regulation for all comparisons. To filter differentially expressed genes we used the following thresholds: fdr <5% and fold change greater 2 or less than 1/2.

**Figure 3:**
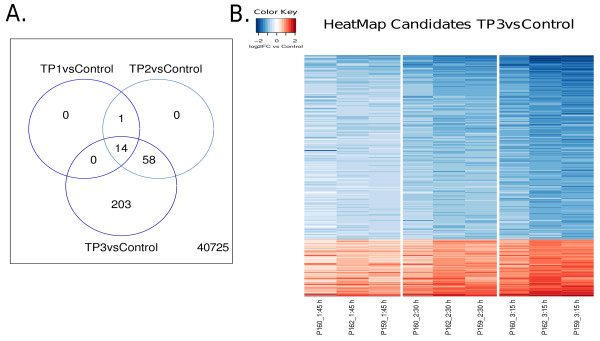
**Differentially Expressed Genes**. (**A**) shows a Venn diagram obtained from all comparisons. Numbers in the circles indicate the amount of overlapping genes between lists of differentially expressed genes. The heatmap in (**B**) shows log2-fold changes for all significant DEGs selected from comparison TP3 vs Control. Red colors indicate over-represented and blue colors under-represented genes in comparison to the corresponding control samples.

To establish a relationship between regulation of genes and increased thermal degradation, a Venn diagram, shown in Figure 3A, summarizes all comparisons. The Venn diagram shows that, with one exception, all candidates between TP1 vs Control and TP2 vs Control are included in the significant DEGs of TP3 vs Control. The number of differentially expressed genes increases with progressive RNA degradation. Given this subset relation, we performed all further analyses with significant DEGs from TP3 vs Control, because this list contains nearly all other DEGs. All significant DEGs of TP3 vs Control are shown in a heatmap in Figure 3B including individual log2-fold-changes for all samples. It can be seen that for the majority of DEGs, the level of regulation is increasing proportionally with the RNA degradation level.

**Figure 4:**
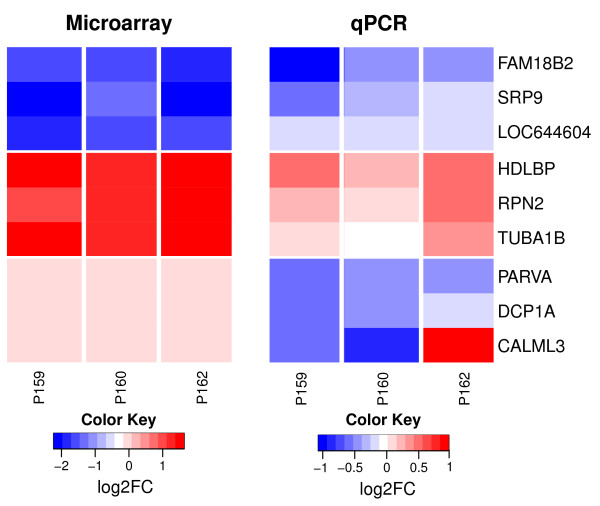
**Comparison of Microarray and qPCR results for candidate genes**. Heatmaps from microarray (left side) and qPCR (right side). Colors represent relative differences between TP3 vs. Control. 9 genes from 3 groups of representation (from top to bottom: over-represented, under-representation and normally-represented in the microarray data) are shown.

Validation of the microarray data was performed by qPCR using three representative candidates of under-represented, over-represented and normally-represented genes from the control group and TP3. Therefore, the degradation experiment was repeated, i.e. RNA from the same three patients was degraded again. To compare the expression levels fold changes between the two time points were assessed as illustrated in Figure 4 showing similar patterns of over- and under-representation. Only the normally-represented group showed a slight tendency to be more degraded in the qPCR experiments than in the microarray results. This might be explained by a higher stability of the house-keeping genes that were used in the qPCR experiments or by the fact that qPCR reveals the general trend of degradation, also observed on the microarray but removed due to the normalization procedure. Generally the direction of change remains consistent for the two groups of over-represented and under-represented genes.

Interestingly, the relative differences in the qPCR are smaller compared to the microarray data. This finding might be explained by the different techniques of reverse transcription that is carried out by random priming for the qPCR and by oligo-dT for the microarray experiments. Since random priming can occur at any position within the RNA and is not restricted to the 3' end subsequent PCR will be successful if the entire amplicon is contained within a single cDNA molecule. This is dependent on the size of the RNA fragments present during RT and the size of the amplicon during qPCR but the absolute position of the amplicon within the RNA is not critical. This is different for microarray experiments where cDNA synthesis is initiated at the 3' end of the mRNA. As a result, qPCR may to be more robust than DNA-chip analyzes.

To functionally characterize the genes affected by RNA degradation, we searched for over-represented Gene Ontology terms. The analysis to detect enriched Gene Ontology (GO) terms was carried out separately for under- and over-represented RNAs to identify groups of genes with similar functions. The results of significant GO terms (p-value < 0.01) are listed in Table 3.

**Table 3:** Gene Ontology Analysis

Under-represented Genes										
**Ontology**	**Rank**	**GO.ID**	**Term**	**Annotated**	**Significant**	**Expected**	**Rank in classic**	**classic**	**elim**	**weight**

BP	1	GO:0006099	tricarboxylic acid cycle	41	6	0.23	1	9.7e-08	9.7e-08	9.7e-08
	2	GO:0045900	negative regulation of translational elongation	6	3	0.03	10	3.3e-06	3.3e-06	3.3e-06
	3	GO:0046500	S-adenosylmethionine metabolic process	7	3	0.04	12	5.7e-06	5.7e-06	5.7e-06
	4	GO:0006446	regulation of translational initiation	70	5	0.39	19	4.3e-05	4.3e-05	4.3e-05
	5	GO:0006614	SRP-dependent cotranslational protein targeting to membrane	13	3	0.07	20	4.5e-05	4.5e-05	4.5e-05
	6	GO:0007021	tubulin complex assembly	5	2	0.03	31	0.00030	0.00030	0.00030
	7	GO:0046498	S-adenosylhomocysteine metabolic process	5	2	0.03	32	0.00030	0.00030	0.00030
	8	GO:0030091	protein repair	6	2	0.03	33	0.00045	0.00045	0.00045
	9	GO:0006425	glutaminyl-tRNA aminoacylation	1	1	0.01	51	0.00554	0.00554	0.00554
	10	GO:0006430	lysyl-tRNA aminoacylation	1	1	0.01	52	0.00554	0.00554	0.00554
	11	GO:0043393	regulation of protein binding	25	2	0.14	54	0.00839	0.00839	0.00839

MF	1	GO:0000104	succinate dehydrogenase activity	8	5	0.04	1	2.4e-10	2.4e-10	2.4e-10
	2	GO:0005047	signal recognition particle binding	7	3	0.04	3	5.4e-06	5.4e-06	5.4e-06
	3	GO:0045182	translation regulator activity	211	8	1.15	4	2.1e-05	0.27124	2.1e-05
	4	GO:0008312	7 S RNA binding	14	3	0.08	5	5.5e-05	5.5e-05	5.5e-05
	5	GO:0008119	thiopurine S-methyltransferase activity	3	2	0.02	7	8.8e-05	8.8e-05	8.8e-05
	6	GO:0003924	GTPase activity	328	8	1.79	8	0.00044	0.00044	0.00044
	7	GO:0004719	protein-L-isoaspartate (D-aspartate) O-methyltransferase activity	10	2	0.05	16	0.00129	0.00129	0.00129
	8	GO:0005525	GTP binding	592	10	3.22	19	0.00150	0.00150	0.00150
	9	GO:0009055	electron carrier activity	349	7	1.90	22	0.00304	0.00304	0.00304
	10	GO:0004819	glutamine-tRNA ligase activity	1	1	0.01	26	0.00544	0.00544	0.00544
	11	GO:0004824	lysine-tRNA ligase activity	1	1	0.01	27	0.00544	0.00544	0.00544
	12	GO:0003723	RNA binding	1269	17	6.91	10	0.00052	0.00680	0.00680

CC	1	GO:0005786	signal recognition particle, endoplasmic reticulum targeting	15	4	0.08	3	1.2e-06	1.2e-06	1.2e-06
	2	GO:0005785	signal recognition particle receptor complex	7	3	0.04	5	6.0e-06	6.0e-06	6.0e-06
	3	GO:0005853	eukaryotic translation elongation factor 1 complex	17	3	0.10	11	0.00011	0.00011	0.00011
	4	GO:0002079	inner acrosomal membrane	4	2	0.02	13	0.00019	0.00019	0.00019
	5	GO:0042589	zymogen granule membrane	5	2	0.03	16	0.00031	0.00031	0.00031
	6	GO:0008290	F-actin capping protein complex	7	2	0.04	24	0.00065	0.00065	0.00065
	7	GO:0005743	mitochondrial inner membrane	391	11	2.20	6	1.3e-05	1.3e-05	0.00263
	8	GO:0044444	cytoplasmic part	6340	68	35.62	1	1.5e-09	0.01111	0.00499
	9	GO:0045273	respiratory chain complex II	3	2	0.02	10	9.4e-05	9.4e-05	0.00558
	10	GO:0005759	mitochondrial matrix	295	6	1.66	30	0.00652	0.00652	0.00652

**Over-represented Genes**										

Ontology	Rank	GO.ID	Term	Annotated	Significant	Expected	Rank in classic	classic	elim	weight

BP	1	GO:0018279	protein amino acid N-linked glycosylation via asparagine	20	2	0.02	1	0.00013	0.00013	0.00013
	2	GO:0006414	translational elongation	253	3	0.22	3	0.00127	0.00127	0.00127
	3	GO:0000722	telomere maintenance via recombination	2	1	0.00	5	0.00173	0.00173	0.00173
	4	GO:0009446	putrescine biosynthetic process	2	1	0.00	6	0.00173	0.00173	0.00173
	5	GO:0051258	protein polymerization	106	2	0.09	8	0.00374	0.00374	0.00374
	6	GO:0008295	spermidine biosynthetic process	6	1	0.01	9	0.00519	0.00519	0.00519
	7	GO:0007094	mitotic cell cycle spindle assembly checkpoint	8	1	0.01	11	0.00691	0.00691	0.00691
	8	GO:0007018	microtubule-based movement	157	2	0.14	13	0.00802	0.00802	0.00802
	9	GO:0015937	coenzyme A biosynthetic process	10	1	0.01	15	0.00863	0.00863	0.00863

MF	1	GO:0004579	dolichyl-diphosphooligosaccharide-protein glycotransferase activity	19	2	0.02	1	0.00014	0.00014	0.00014
	2	GO:0008783	agmatinase activity	2	1	0.00	3	0.00187	0.00187	0.00187
	3	GO:0005198	structural molecule activity	1149	5	1.08	4	0.00359	0.13592	0.00359
	4	GO:0004594	pantothenate kinase activity	7	1	0.01	6	0.00654	0.00654	0.00654
	5	GO:0003720	telomerase activity	8	1	0.01	7	0.00747	0.00747	0.00747

CC	1	GO:0008250	oligosaccharyl transferase complex	20	2	0.02	1	0.00013	0.00013	0.00013
	2	GO:0005840	ribosome	407	3	0.35	13	0.00493	0.00493	0.00493

The GO analysis was applied to differentially expressed genes from the comparison TP3 vs. Control. The number of genes belonging to a GO term represented on the microarray (annotated), the number genes in the analyzed gene list (significant) and the expected number of genes by chance are given. The **upper part **of the table shows GO terms that are highly significant for under-represented genes (p-value < 0.01); the **lower part **indicates terms that are highly significant for over-represented genes. Calculated p-values for each category from three similar methods (classic, elim, weight) are shown. Each table is separated into three different parts representing the following ontologies: Biological Process (BP), Molecular Function (MF) and Cellular Component (CC).

Although some significant GO terms exist, no relation to known RNA degradation processes or to pathways that are initiated during apoptosis could be found. The absence of these findings clearly shows that changes of the expression profile are generated at the RNA level and not by de-novo transcription, as expected from the experimental design.

As can be seen by this analysis, we do not observe a global effect of RNA degradation on gene expression. RNA degradation seems to have a significant influence only on a fairly small number of genes. If such a global effect of RNA degradation does indeed exist, we do not observe it in the measured gene expression profiles. Indeed it is possible that such an effect is removed by the normalization routine that we use. Note that, by the nature of the hybridization experiments, microarrays produce only a relative measure of gene expression and normalization between the experiments is therefore essential to make the expression intensities comparable. This fact is also necessary to explain the observed over-represented probes (i.e. 65 out of 41,000). These are likely to reflect genes that are relatively less severely affected by RNA degradation than the majority of the measured mRNAs. Therefore, on the other hand the observed under-represented probes (i.e. 210 out of 41.000) reflect genes which are relatively more greatly affected by RNA degradation than the mRNAs of most other genes. This might mean that their mRNAs are less stable. Therefore, in analyses comparing patient samples with varying RNA quality, one should be careful with the interpretation of results when observing these genes as dysregulated.

### Characterization of sequence features in differentially expressed genes

The RNA degradation process is not entirely explained, yet. On the one hand it is postulated, that the process starts from the 5' end [3,24,25], on the other hand a more random mechanism including endonucleases activity has to be expected. This becomes evident interpreting the bioanalyzer data. If the mRNA would be degraded only from one side a broader peak within the 28 S and 18 S peak would be observable. For both assumptions it could be hypothesized that probes which are closer to the 3' end should be efficiently hybridized and be more stable than those located closer to the 5' end. This is also accounted for by the fact that the Agilent microarray probes are predominantly located near the 3' end of the mRNA but also due to the important step of reverse transcription. In case of random degradation incomplete cDNA synthesis becomes more and more probable by the increases of the distance between 3' end (location of poly-A-tail that serves as starting point) and the mRNA sequence that later serves as the probe.

To analyze the effect of oligo positions on expression measurements, we determined the relative positions of Agilent's 60 mer probes within the best matching transcripts via Blast against all available NCBI RefSeq Human transcripts.

**Figure 5:**
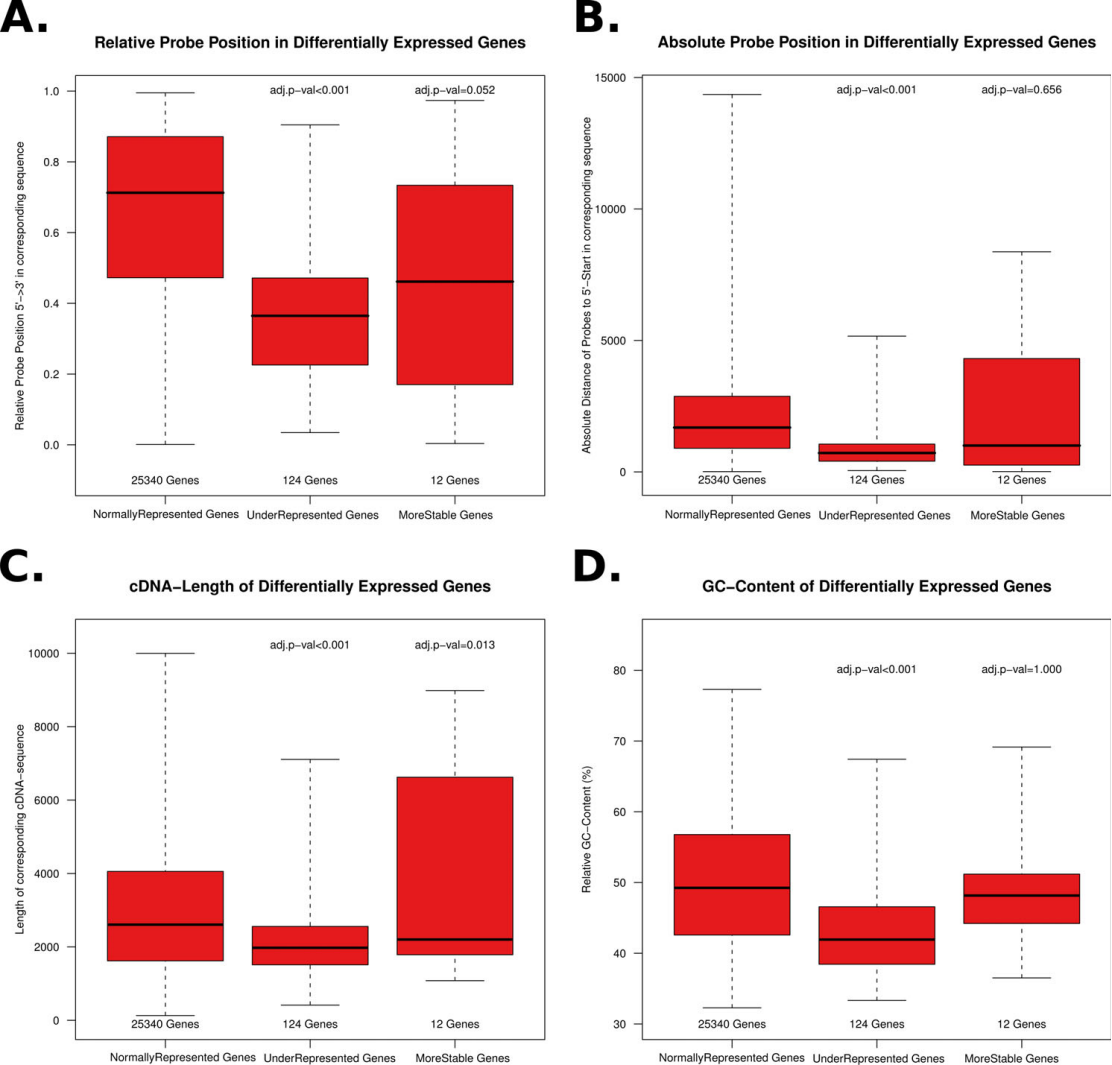
**Characterization of sequence features**. Comparison of sequence features for all genes from the following groups: normally-represented, under-represented and over-represented or more stable genes in TP3 vs Control. The numbers on top indicate the significance levels. The p-values were obtained using the two-sample Wilcoxon test and are Bonferroni corrected. The numbers at the bottom indicate the quantity of genes belonging to the respective group. (**A**) compares the relative probe positions in the corresponding cDNA sequences. (**B**) shows the absolute distance of the probe to the cDNA 5' end. (**C**) displays the effect of absolute cDNA lengths. (**D**) shows the differences in GC content between the explored groups.

The comparison of oligo positions between DEGs and all other probes shows significant differences. The relative positions of probes from under-represented genes are shifted to the 5' region, while probes of normally-represented genes are closer to the 3' end, as can be seen in Figure 5A. This observation is further underlined by looking at the absolute distance between 5' end and probe start. We observed that the distance to the 5' end of probes from under-represented genes is significantly smaller than the distance of probes from normally-represented genes (see Figure 5B). Similarly probes in the under-represented group are further away from the 3' end, see Additional file 1, Figure S1. This is consistent with the comparison of the supposed mRNA lengths between the different groups of genes. Here, we also found significant differences between under-represented and normally-represented genes. Figure 5C shows that the mRNA-lengths of under-represented genes are significantly shorter (Bonferroni corrected p-value from two-sample Wilcoxon test was <0.001). This might be due to the degradation mechanisms that result in a higher probability of loss of detection. However, it does not mean that short RNAs are degraded quicker or more prone to degradation.

In contrast, the genes that appear to be more stable are very long. We consider this over-representation to be due to a more severe degradation of loss of the remaining genes. This means the over-represented genes are very stable genes, even more stable than the normally-represented group, i.e. seemingly not-differential genes that in fact follow a general trend of degradation and reduction of the RNA levels. This is however, not relevant for differential gene expression as the gene expression levels are normalized to the total amount of RNA.

To validate the microarray results, qPCR was performed using three representative candidates of the over-represented, under-represented and normally-represented genes. Although the fold change from qPCR that was used to assess the difference between the two time points was lower than that from microarray analyses, the direction of regulation could be confirmed for the over- and under-represented genes. The result seems to be conflicting for the normally-represented genes. However, this difference can be attributed to the different normalization procedures on the array, i.e. based on the overall mean, or in the qPCR data, i.e. based on housekeeping genes, as well as on the the different mechanisms of reverse transcription that is used for qPCR and expression arrays. While the decrease of signal intensity over the entire array between the two time points is not visible within the normalized array data they are apparent using qPCR. The nucleotide composition is often used to determine stability or instability of nucleic acids. The GC content especially is an indicator of stability of secondary structures. The results of comparisons according to the GC content are given in Figure 5D. Under-represented genes have a significantly lower GC content than the normally-represented genes.

In summary, we showed that different sequence features, such as probe position, mRNA-length or GC content, play a role in the perceived effects of RNA degradation when using microarrays for gene expression profiling.

## Discussion

Decoding the transcriptome is a major goal in the process to gain a better understanding of the underlying mechanisms of a disease and the potential for discovering therapeutic targets. Using microarray technology, high-throughput expression data for the whole genome can be generated within a short period of time. This gives us the unique possibility of screening a high number of patient samples for a massive number of features. Nevertheless, working with clinical samples is challenging. It is important in this kind of investigation to have a high degree of standardized procedures (e.g. storage of tissue or isolation of RNA), as a technical bias might otherwise arise that would override the biological differences of the samples. Furthermore, tissue quality is a serious concern as well as RNA quality. RNA degradation always takes place in the tissue that is removed and stored for later processing. However, this process is not only influenced by the time between removing the specimen and the removal of the tissue specimen, but rather by the time between removal of the specimen and the disruption of the blood supply that depends on the type of tissue that is removed. The level of degradation can vary tremendously, which leads to the question of how far degradation influences the results of gene expression screens.

To assess this influence, we simulated RNA degradation in vitro by using heat treatment of patient samples. We believe that this approach provides data that is more relevant to our approach of gene expression profiling. However, it remains unclear to which extent this resembles the in-vitro situation or changes during tissue preparation. Since in previous studies, tissue was treated to achieve degradation [4-6]. Those studies have to deal with the problem of differences due to functional changes in living cells such as apoptotic changes for example. We therefore used heat induced degradation that has previously been used in [26].

Moreover, degradation of mRNA itself instead of degradation of the tissue after removal is closer to the clinical routine. Firstly because in the clinical practice of taking rectal cancer biopsies, for example, the tissue is taken from the patients and transferred into RNA conserving fluid within less than 20 seconds. Secondly, when using the same mRNA for later analysis, the homogeneity of the investigated material is higher than when taking different tissue samples. For normal tissue, the problem of heterogeneity might be negligible, but in cancer, that is well known for its heterogeneous set-up, applying different tissue samples introduces the risk that the heterogeneity is behind the expression differences, and not the degradation.

To standardize the degradation state of the samples, we used the RNA integrity number (RIN). As expected, the RIN decreased as the time of heat treatment increased. The degradation showed a similar trend that was highly correlated for all patient samples.

The main purpose of our analysis was to investigate to which extent RNA degradation limits the comparability of gene expression data derived from rectal cancer biopsies from different patients. Although quality thresholds of 6 or 7 were set in the past [4,5] these results have to be interpreted carefully since non-cancerous tissue (e.g. rat liver) or different conservation methods (fresh frozen) were applied. Furthermore, as already discussed, different biopsies from patients with rectal cancer previously degraded in a time-dependent manner were analyzed.

In a preliminary test, RNA was exposed to different temperature levels. No degradation occurred at room temperature and only very little at 45°C. Additionally, very different levels of degradation were retrieved compared to the setup at 60°C. To get comparable degradation results, we therefore choose four time points that showed RIN differences of up to 4 levels (P159; 0:00 h RIN 9 and 3:15 h RIN 4.7). Interestingly, PCA as well as clustering analysis revealed very high similarity of samples from the same patient and samples from different patients did not cluster together revealing their high biological divergence.

Although biology was the dominating effect of both PCA and cluster analysis in our case, certain gene expression differences were found and became more and more evident the more the RNA was degraded. Apart from an overlap of genes between the samples at comparable degradation time points, genes that were differentially expressed at an early degradation time point reappeared as differentially expressed at the later time points as well, indicating that changes observed on gene expression in all three comparisons originated exclusively from the degradation. Furthermore, the over- or under-expression of a given gene was constant throughout the entire experiment. Though these changes were not derived from specific regulatory processes we considered these changes not as an expression itself but rather as a representation of the genes.

Since we considered these changes in dysregulation as specific, we looked more closely at these genes trying to find functional similarities. Using Gene Ontology, a few processes were found but none of them could be put into the context of degradation.

These results suggest that degradation activated by heating is closely and more related with the nature of mRNA degradation than with the activation of a pathway involved in a specific biological process. We identified short mRNAs to be under-represented that might be due to the higher probability of affecting sequences that are important for detection by microarray.

A third stability marker for a more stable gene expression was the position of the complementary probe which was spotted within the gene. Accordingly, genes with probes that were designed to bind closer to the 3' end were found to be more stable.

Investigating the length distribution and probe positions of the most under-represented genes, we found that short genes especially were affected, as well as those with a short distance between the 5' end and the probe binding sequence.

This finding is based on two essential degradation mechanisms. First the degradation from the 5' end but second and more obvious a random degradation. In that case affection of the mRNA between the 3' end (where the Poly-A-tail is located and that is used for reverse transcription in Agilent arrays) and the sequence that later binds to the array probe is more probable the longer the distance between the probe and the 3' end, or the shorter the distance between the probe and the 5' end, respectively. In this context, we can also assume that degradation by heating is less effective for longer transcripts with a high GC content.

However, these results have some limitations. The platform used within these analyses has to be taken into consideration. Apart from different methods of reverse transcription as for example the use of oligo-dT primers versus random hexamers the platform itself might play a major role. While the probes that are spotted on the Agilent 44 K array used here are 60 mer long those from the Affymetrix chip for example are only 25 bp long. This difference might strongly influences the binding characteristics to the microarray, especially when degraded RNA is used. Furthermore we investigated a small group of different rectal cancers. The results, that implicit a much higher difference based on biology than on degradation might only hold true as long as such heterogeneity within the investigated samples can be found.

## Conclusions

In summary, RNA degradation is an important process that might hinder the analysis of gene expression profiles from patient samples. Patient samples are valuable in clinical studies; therefore, we have to assess which samples, with respect to RNA quality, can be used in later analysis and which samples cannot be used. In conclusion, RNA of different quality can be used to perform gene expression analysis due to a much higher biological variance, like in our case compared to the effect that is imposed by degradation of RNA. Nevertheless there are few genes that are detected with lower signals on the microarray due to degradation, especially very short ones and those with the probe binding site close to the 5' end. As a consequence of degradation, not only are the short genes affected in their differential expression, but additionally the very long genes appear different due to the fact that they are less affected by degradation. Therefore, these genes should be excluded from gene expression analysis when working with degraded RNA.

## Competing interests

The authors declare that they have no competing interests.

## Authors' contributions

JG, MG and TB proposed the concept and design of this study. BMG provided the clinical samples. JG prepared the clinical samples. LO and GSR performed the microarray studies. The qPCR validation was done by PJ and GE. LO and KJ performed the data analysis. LO, JG, GSR and TB wrote the manuscript. All authors contributed in discussions and approved of the final manuscript.

## Pre-publication history

The pre-publication history for this paper can be accessed here:

http://www.biomedcentral.com/1755-8794/3/36/prepub

## Supplementary Material

Additional file 1 **Supplementary Figure S1**. The absolute distance of the probe to the cDNA 3' end for all genes from the following groups is shown: normally-represented, under-represented and over-represented genes in TP3 vs Control. The numbers on top indicate the significance levels. The p-values were obtained using the two-sample Wilcoxon test and are Bonferroni corrected. The numbers at the bottom indicate the quantity of genes belonging to the respective group. Click here for file

Additional file 2 **Supplementary Figure S2**. We checked the technical reproducibility of Agilent microarray results using the 3 patient samples analyzed here. We investigated the robustness of gene expression profiles in dependence of: 1. the experimenter (E); or 2. repeating the labelling (L); or 3. repeating the hybridization (H); or 4. using different washing methods (W). These types of technical replicates where highly correlated and clustered together. Supplementary Figure 2 shows pairwise correlations between all samples of patients. The elements of the matrix in the visualization are colored by Pearson's correlation coefficient values with deeper colors indicating higher positive (blue) correlations. The heatmap is flanked by clustering dendrograms showing the similarity between samples in a hierarchical approach. Click here for file
