# Supplementary figures and images for: Impact of RNA degradation on gene expression profiling
Source: BMC Med Genomics. 2010 Aug 9;3:36. doi: 10.1186/1755-8794-3-36 (PMC2927474; doi:10.1186/1755-8794-3-36)

# Absolute Probe Position in Differentially Expressed Genes

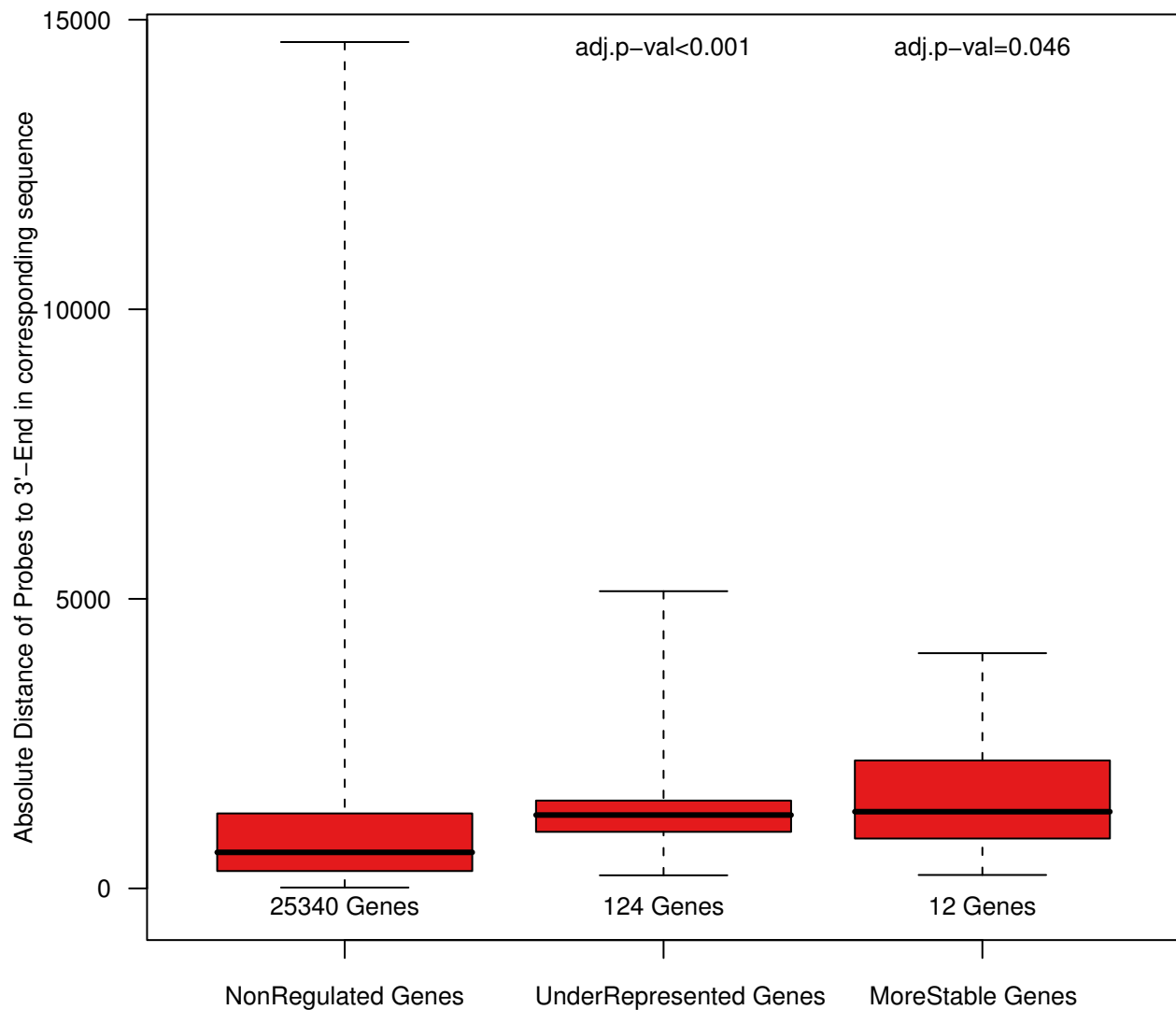

Supplement: Additional file 1 — Supplementary Figure S1. The absolute distance of the probe to the cDNA 3' end for all genes from the following groups is shown: normally-represented, under-represented and over-represented genes in TP3 vs Control. The numbers on top indicate the significance levels. The p-values were obtained using the two-sample Wilcoxon test and are Bonferroni corrected. The numbers at the bottom indicate the quantity of genes belonging to the respective group. [file 1755-8794-3-36-S1.PDF]

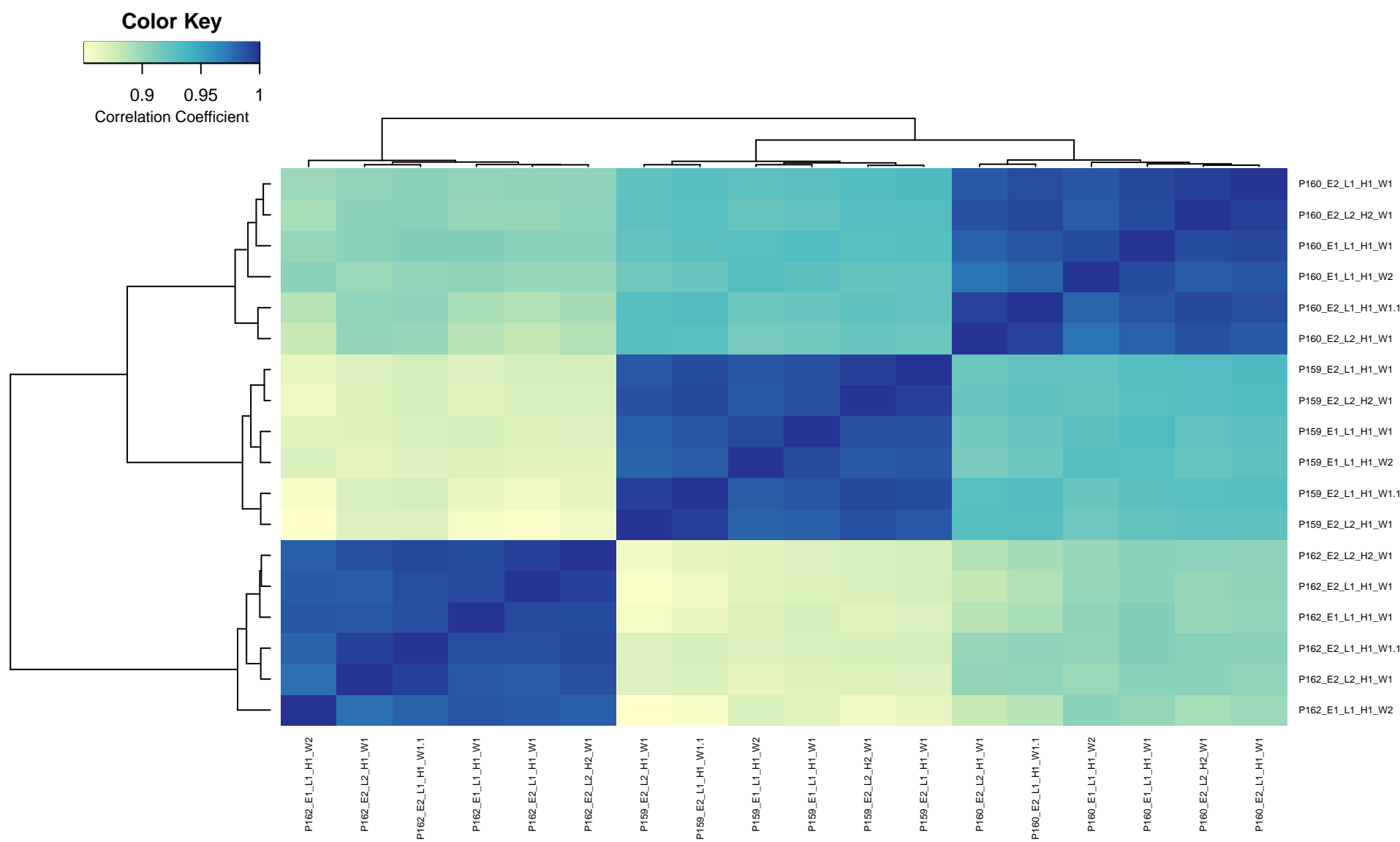

Supplement: Additional file 2 — Supplementary Figure S2. We checked the technical reproducibility of Agilent microarray results using the 3 patient samples analyzed here. We investigated the robustness of gene expression profiles in dependence of: 1. the experimenter (E); or 2. repeating the labelling (L); or 3. repeating the hybridization (H); or 4. using different washing methods (W). These types of technical replicates where highly correlated and clustered together. Supplementary Figure 2 shows pairwise correlations between all samples of patients. The elements of the matrix in the visualization are colored by Pearson's correlation coefficient values with deeper colors indicating higher positive (blue) correlations. The heatmap is flanked by clustering dendrograms showing the similarity between samples in a hierarchical approach. [file 1755-8794-3-36-S2.PDF]
